# Improvement of the Interface between the Lithium Anode and a Garnet-Type Solid Electrolyte of Lithium Batteries Using an Aluminum-Nitride Layer

**DOI:** 10.3390/nano12122023

**Published:** 2022-06-12

**Authors:** Wen Jiang, Lingling Dong, Shuanghui Liu, Bing Ai, Shuangshuang Zhao, Weimin Zhang, Kefeng Pan, Lipeng Zhang

**Affiliations:** 1 School of Chemistry and Chemical Engineering, Shandong University of Technology, Zibo 255049, China; wengejang@126.com (W.J.); donglingling202107@163.com (L.D.); shuanghuiliu2020@163.com (S.L.); aibing@sdut.edu.cn (B.A.); wmzhang@sdut.edu.cn (W.Z.); 2 School of Materials and New Energy, South China Normal University, Shanwei 516600, China; 20219207@m.scnu.edu.cn

**Keywords:** LLZTO, solid-state electrolytes, lithium/electrolyte interface, anode interface, lithium-ion battery

## Abstract

The next generation of all-solid-state batteries can feature battery safety that is unparalleled among conventional liquid batteries. The garnet-type solid-state electrolyte Li_7_La_3_Zr_2_O_12_ (LLZO), in particular, is widely studied because of its high Li-ion conductivity and stability in air. However, the poor interface-contact between Li and the electrolyte (garnet) severely limits the development of solid electrolytes. In this study, we synthesize cubic phase Li_6.4_La_3_Zr_1.4_Ta_0.6_O_12_ (LLZTO) using a secondary sintering method. In addition, a thin aluminum nitride (AlN) layer is introduced between the metal (Li) and the solid electrolyte. Theoretical calculations show that AlN has a high affinity for Li. Furthermore, it is shown that the AlN coating can effectively reduce the interface impedance between Li and the solid electrolyte and improve the lithium-ion transport. The assembled symmetric Li cells can operate stably for more than 3600 h, unlike the symmetric cells without AlN coating, which short-circuited after only a few cycles. The hybrid solid-state battery with a modified layer, which is assembled using LiFePO_4_ (LFP), still has a capacity of 120 mAh g^−1^ after 200 cycles, with a capacity retention rate of 98%. This shows that the introduction of an AlN interlayer is very helpful to obtain a stable Li/solid-electrolyte interface, which improves the cycling stability of the battery.

## 1. Introduction

The widespread use of electronic devices and the growing popularity of electric vehicles led to higher demands for the energy density of batteries. Unfortunately, a higher energy-density also tends to decrease battery safety [1,2,3,4]. Traditional liquid lithium-ion batteries use flammable organic electrolytes and diaphragms, which are prone to lithium dendrites piercing the diaphragm and causing a short-circuit inside the battery that can lead to a thermal runaway, fire, and even explosion. It is critical that battery safety is maintained when the energy density of the battery improves. Unfortunately, this is not an easy problem to solve, and this limits the development of conventional lithium-ion batteries [5,6,7,8,9,10]. All-solid-state batteries (ASSBs) can, in principle, solve the safety problem of conventional lithium-ion batteries by using a solid electrolyte instead of the organic diaphragm and liquid electrolyte of conventional liquid batteries. Moreover, ASSBs have several advantages such as high energy-density and excellent cycling performance [11,12,13]. Currently, all-solid-state batteries are intensively studied because they are promising candidates for the next generation of battery technology [14,15].

The key characteristic of ASSBs batteries is, of course, the solid-state electrolyte. Currently, solid state electrolytes are mainly divided into organic polymer solid-state electrolytes (OSSEs) and inorganic solid-state electrolytes (ISSEs) [16,17]. The OSSEs are widely studied. They include polyethylene oxide (PEO) [18], polyvinylidene fluoride (PVDF) [19], poly(vinylidene fluoride-co-hexafluoropropylene) (PVDF-HFP) [20], polyacrylonitrile (PAN) [21], and polymethyl methacrylate (PMMA) [22]. OSSEs usually have excellent processability, flexibility, safety, and good interface contact with electrodes [23,24]. However, the ionic conductivity of OSSEs is generally low (<10^−4^ Scm^−1^), both thermal and electrochemical stability is poor, and the suppression of lithium dendrites is unsatisfactory, which seriously limits their development [25].

ISSEs, on the other hand, include mainly lithium phosphorus oxynitride (LiPON) [26,27], perovskite [28], sulfide [29], and garnet [30]. LiPON solid-state electrolytes show excellent overall performance, but the required special sputtering process limits their large-scale development [31]. Perovskite-based structural materials have higher ionic conductivity at low temperatures than conventional electrolytes [32]. Their biggest disadvantage is that Ti^4+^ is reduced when it comes into contact with lithium [33]. Sulfide-based solid-state electrolytes have relatively high lithium-ion conductivity and low activation energy [34]. However, they are highly sensitive to air and prone to producing toxic H_2_S. In addition, sulfide solid electrolytes are prone to react with lithium, which limits the doping of the lithium anode [35].

Compared to the solid-state electrolytes above, the garnet electrolyte Li_7_La_3_Zr_2_O_12_ (LLZO) has a higher ionic conductivity, a wider electrochemical stability window, and better stability in air [36], which makes it a promising candidate electrolyte for ASSBs. Although the LLZO electrolyte has many advantages, the ionic conductivity of the tetragonal phase is low at room temperature, and the cubic phase with its high ionic conductivity is difficult to stabilize at room temperature [37]. During the calcination process, doping with Al, Nb, Ta, and other elements is an effective method to stabilize the cubic phase at room temperature [38]. Secondly, the problem of poor contact between the garnet solid electrolyte and lithium limits the development of solid electrolytes. Typically, a buffer layer made of metals such as Au [39,40], Mg [41], Ge [42], Nb [39], Sn [43], as well as metal-oxide layers such as ZnO [44], SnO_2_ [45], Al_2_O_3_ [46,47], can be introduced between the Li and the LLZO. This buffer layer can effectively reduce the interface impedance between Li and LLZO and strengthen the interface contact between Li and LLZO. It was previously thought that polishing LLZO in an inert gas would be effective in improving the interfacial contact between LLZO and the Li metal, but recent studies have shown that mechanical polishing actually creates an inert layer on the LLZO surface, and that short etching of LLZO with HCl in air can effectively remove the inert layer and thus release the intrinsic electrochemical activity of LLZO [48,49,50].

Moreover, Li_9_Al_4_ sites can be generated, in situ, on AlN nanoclusters with Li_3_N to promote rapid migration of Li^+^ ions and uniform plating/exfoliation [51,52]. In this study, we obtain Ta-doped Li_6.4_La_3_Zr_1.4_Ta_0.6_O_12_ (LLZTO) using a secondary sintering method, and we successfully introduce a homogeneous AlN layer between Li and LLZTO to improve the affinity of the Li electrode with the solid electrolyte. Theoretical calculations show that AlN has a high affinity for Li, and AlN is also wettable for LLZTO. The experimental results show that the AlN coating confirms that not only can it effectively reduce the interface impedance between Li and solid electrolyte, but it can also facilitate the lithium-ion transport. The assembled symmetric Li cells can operate stably for more than 3600 h, unlike the symmetric cells without the AlN coating (which short-circuit after only a few cycles). The hybrid solid-state battery with the modified layer, which is assembled using LiFePO_4_ (LFP), still has a capacity of 120 mAh g^−1^ after 200 cycles, with a capacity retention rate of 98%. This shows that the method of introducing an AlN interlayer is very effective to construct a stable Li solid-electrolyte interface and improve the cycling stability of the battery.

## 2. Materials and Methods

### 2.1. Preparation of Garnet LLZTO Electrolytes

Cubic garnet Li_6.4_La_3_Zr_1.4_Ta_0.6_O_12_ (LLZTO) electrolyte was prepared using a conventional solid-state reaction. Stoichiometric amounts of La_2_O_3_ (Aladdin Inc., Shanghai, China, 99.99% purity), ZrO_2_ (Aladdin Inc., Shanghai, China, 99.99% purity), Ta_2_O_5_ (Aladdin Inc., Shanghai, China, 99.99% purity), 1.2 wt% Al_2_O_3_ (Aladdin Inc., Shanghai, China, 99.99% purity), and 15 wt% excess of LiOH (Aladdin Inc., Shanghai, China, 98% purity) were used to compensate the loss of Li during the calcination. The starting materials were mixed with isopropanol and placed in a planetary ball-mill with small zirconia balls, at 600 rpm for 12 h for homogenization. The precursor powder was sintered in air at 900 °C for 12 h. Subsequently, the powder was ground via ball-milling for another 12 h at 600 rpm. Finally, the powder was pressed into 15 mm pellets under a pressure of 18 MPa and sintered at 1170 °C for 12 h in a muffle furnace. The pellets were covered by the mother powder to reduce lithium loss during sintering. The sintered pellets were mirror-polished well with 800, 1200, 1500, and 2000 grit SiC sandpaper, ultrasonically cleaned, dried, and stored in an argon-filled glovebox until further use.

### 2.2. Preparation of the AlN Mixed Interlayer

**Figure 1:**
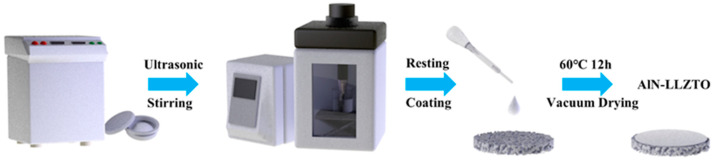
Schematic illustration of AlN coated LLZTO.

The AlN (Aladdin Inc., Shanghai, China, 99.5% purity) (Supplementary Figure S1) thin films were deposited on the polished LLZTO pellets via manual spot coating. The preparation process is shown in Figure 1. Firstly, AlN powder and PVDF were mixed, with a mass ratio of 9:1, and then ground well. Then, the solvent 1-Methyl-2-pyrrolidinone (NMP) was added and placed in a slurry machine at 2000 rpm for 15 min. The mixed slurry was ultrasonicated to ensure it is dispersed evenly. Finally, the mixed slurry was added, dropwise, to the surface of the LLZTO electrolyte sheet using a disposable pipette. After the slurry had been evenly dispersed, the coated electrolyte sheet was dried in a vacuum dryer at 60 °C for 24 h to obtain AlN-LLZTO. The required material for the symmetric cell was coated on the other side using the same method.

### 2.3. The DFT Method

All calculations were performed using the projector augmented wave (PAW) method in the framework of density functional theory (DFT), as implemented in the Vienna ab-initio Simulation Package (VASP) [53,54]. The generalized gradient approximation (GGA) and Perdew–Burke–Ernzerhof (PBE) exchange functional was used [55,56]. The plane-wave energy cutoff was set to 500 eV. The Monkhorst–Pack method with 2 × 2 × 1 k-meshes was employed for the Brillouin zone sampling of Li, AlN, and LLZTO. For the interface calculations with large supercells, we only needed the 2 × 2 × 1 k-mesh. The convergence criteria for the energy and force calculations were set to 10^−5^ eV∙atom^−1^ and 0.01 eV∙Å^−1^, respectively. S2 provides details of the DFT method.

### 2.4. Assembly of Symmetric Cells and Hybrid Solid-State Full Cells

Symmetric cells and hybrid solid-state full cells were assembled using a standard 2032 cell-battery shell. Symmetric cells were assembled using two lithium disks (~0.6 mm thick and ~10.0 mm in diameter) attached to both sides of the AlN-modified LLZTO pellets (~0.8 mm thick and ~12.0 mm in diameter) to produce a sandwich structure. For comparison, a symmetric cell without the modified layer was assembled at the same time. The LiFePO_4_ (LFP) cathode was made by casting the carbon-coated LiFePO_4_ powder, Ketjen Black (KB), and polyvinylidene fluoride (PVDF), with a weight ratio of 8:1:1 in NMP onto the Al foil. After drying in a vacuum oven at 60 °C, the obtained cathode film was punched into disks with a diameter of 12 mm. A drop of liquid electrolyte (~10 μL, 1.0 mol L^−1^ LiPF_6_ in ethylene carbonate and diethyl carbonate (EC/DEC (volume ratio 1:1)) was added between the LFP cathode and the LLZTO pellets to improve the interface contact between the cathode and the solid electrolyte. The full cell was sealed inside a 2032 coin-battery housing. The battery assembly process was carried out in an Ar-filled glovebox.

Electrochemical impedance spectroscopy (EIS) measurements were performed with a Zennium electrochemical workstation (Zahner Inc., Berlin, Germany), an operating frequency range from 4 MHz to 10 Hz and an amplitude of 10 mV. The ionic conductivity of the LLZTO pellet was tested after sputtering Ag layers on both sides of the pellet, which serve as blocking electrodes. Constant current charge/discharge tests of full batteries were performed at various current densities (e.g., 1 C = 170 mA g^−1^) in the voltage range of 4.0–2.5 V using Neware battery testers. The symmetric cells were tested at 30 °C.

### 2.5. Characterizations

The crystal structures of the samples were examined using X-ray diffraction (Rigaku Inc., Tokyo, Japan) with Cu Kα radiation (λ = 0.15418 nm). The scan range was 10–80° with a scan speed of 10°/min. Raman spectroscopy was performed with a Raman spectrometer (Horiba Inc., Paris, France). The morphology of the samples was observed with a scanning electron microscope (FEI Inc., Hillsboro, OR, USA), equipped with an energy-dispersive spectroscope (EDS).

## 3. Results

### 3.1. Characterization of LLZTO Solid Electrolyte Materials

**Figure 2:**
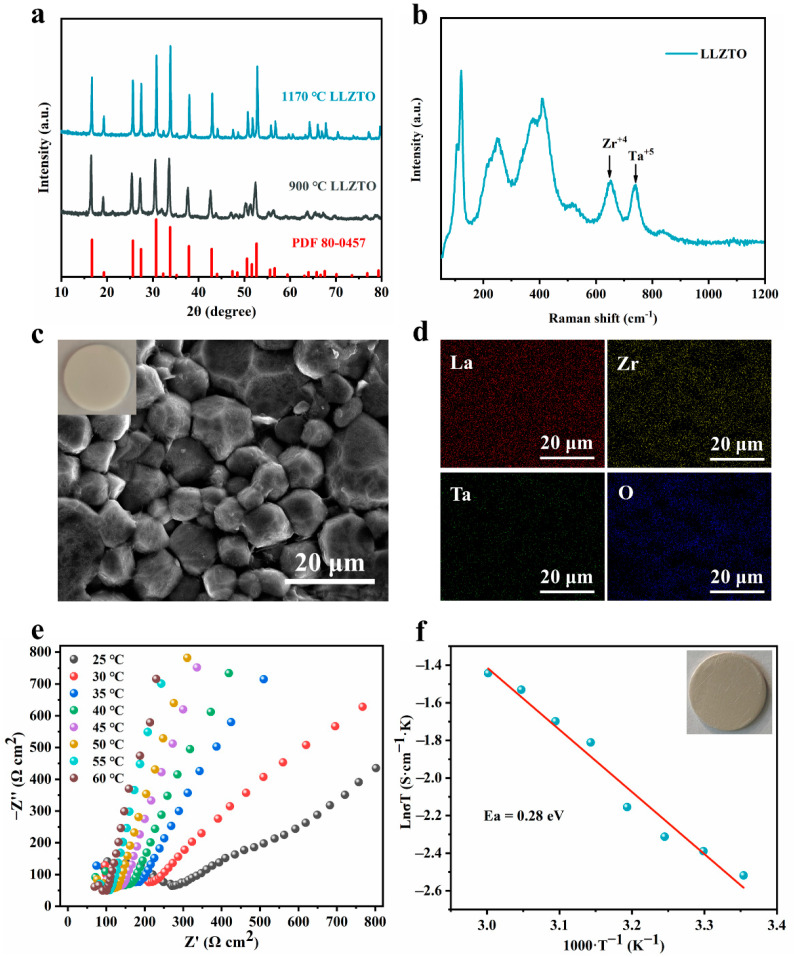
(**a**) The XRD pattern of the as-prepared LLZTO match the cubic structure well; (**b**) Raman spectra of the LLZTO; (**c**) cross-section SEM image of the LLZTO pellet, inset is a photo of an LLZTO pellet; (**e**) EIS profiles of the LLZTO electrolyte at different temperatures in the range 25–60 °C; (**f**) Arrhenius plots of the ionic conductivity for the LLZTO. Inset is a photo of Ag-LLZTO.

The cubic-phase LLZTO solid electrolyte was synthesized using a secondary sintering method. Figure 2a shows the X-ray diffraction (XRD) patterns of the pre-sintered powder at 900 °C and the LLZTO powder prepared by sintering LLZTO dense pellets at 1170 °C. Both diffractograms are consistent with cubic garnet Li_5_La_3_Nb_2_O_12_ (PDF 80-0457), which indicates that the pure cubic LLZO pellets were produced as we expected. The shape of the XRD peak of the sample after sintering at 1170 °C is sharper, which suggests higher crystallinity of the sample. Figure 2b shows the Raman spectrum of the sintered LLZTO sample. The low-frequency region (<300 cm^−1^) vibrational bands can be assigned to the LiO_6_ octahedral unit (96h_Li2_ position), while the middle-frequency region (300–550 cm^−1^) vibrational bending modes can be assigned to the LiO_4_ tetrahedral unit (24d_Li1_ position). The high-frequency region (>550 cm^−1^) bands correspond to the stretching mode of the ZrO_6_ octahedral unit (16a position) [57]. The Raman spectra of tantalum-doped LLZO are consistent with the cubic phase of LLZO garnet reported in the literature [58,59]. The peaks at 625 and 720 cm^−1^ correspond to the band of the stretching mode of the Zr-O bond and the additional band of the Ta-O unit, respectively. The very strong Raman peak, which corresponds to the vibrational mode of CO_3_^−2^ and generally appears at 1090cm^−1^, was not observed in LLZTO. This indicates that the pellets were free from Li_2_CO_3_ [60]. A cross-section SEM image of the sintered pellet is shown in Figure 2c and Figure S3. It can be seen that the particle shape of the sample, which was pre-sintered at 900 °C, is vein-like, and when the calcination temperature was 1170 °C, the grain size increased and showed a dense morphology. Furthermore, it is found that most grains are tightly packed. In some areas, the grain boundaries disappear due to grain amalgamation. The corresponding EDS mappings (Figure 2d) for the elements O, La, Zr and Ta from the cross-section SEM image (Figure 2c) further show that the localization of O, La, Zr, and doping ions Ta was uniformly distributed among the crystal grains. According to the EIS spectra (Figure S3), the semicircle for the high-frequency part comes from the total impedance comprising both bulk and grain boundary resistances. The tail at low frequency is caused by ion-blocking Ag electrodes. The Li-ion conductivity of the LLZTO pellet, which can be derived from the low-frequency intercept, was calculated to be 2.7 × 10^−4^ S cm^−1^ at 25 °C. Figure 2e shows the Nyquist plots for the LLZTO pellet range from 25 to 60 °C. Clearly, the Li-ion conduction of the LLZTO pellet was increased at higher temperatures. The activation energy for Li-ion conduction is 0.28 eV, which was calculated using the Arrhenius equation (S5) and the Arrhenius plots in Figure 2f.

### 3.2. Characterization of AlN Modified LLZTO

**Figure 3:**
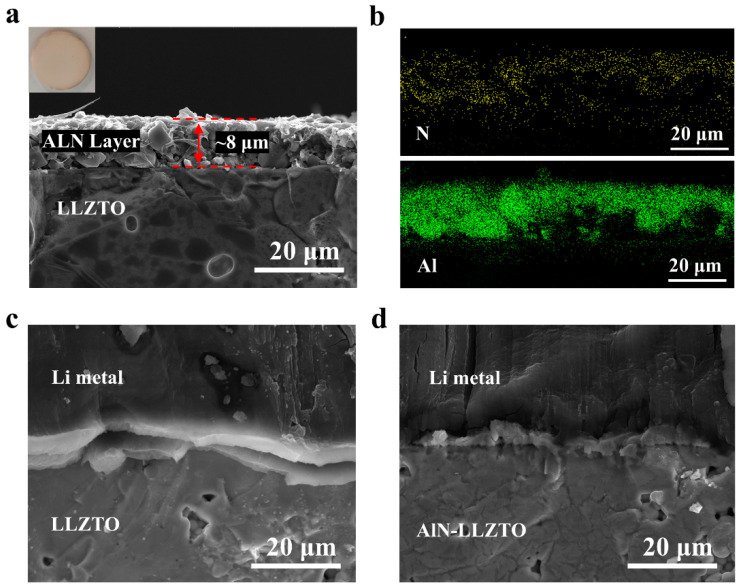
Cross section SEM image of the AlN-LLZTO (**a**), and the corresponding EDS mappings of N and Al (**b**). The inset is a photo of the AlN-LLZTO pellet. Cross-section SEM images of the Li/LLZTO interface (**c**) without- and (**d**) with AlN-interlayer.

Figure 3a shows the cross-section SEM of the as-prepared LLZTO interface with AlN layer. The thickness of the AlN hybrid coating is about 8 µm, and the hybrid coating of AlN and cross-linked PVDF is flexible and adheres effectively to the LLZTO surface. The AlN hybrid coating can wet the LLZTO interface and fill the cavities at the surface of the solid electrolyte, which improves the interface contact between lithium and the solid electrolyte, and it facilitates ion transport. Figure 3b shows the EDS mapping of the modified AlN/LLZTO interface. It indicates that the AlN layer is in close contact with the LLZTO solid electrolyte sheet, and the AlN is distributed uniformly. As shown in Figure 3c, the poor contact between lithium and the non-interface modified LLZTO solid electrolyte leads to significant gaps between the two. The introduction of the AlN interlayer fills the cavities in the surface of the solid electrolyte. As shown in Figure 3d, the lithium metal and LLZTO are in close contact, and no cracks are found. This indicates that the AlN coating effectively enhances the interface contact, and the two-phase interface (now with good contact) can better promote the transport of ions.

### 3.3. Electrochemical Analysis of AlN Modified LLZTO

**Figure 4:**
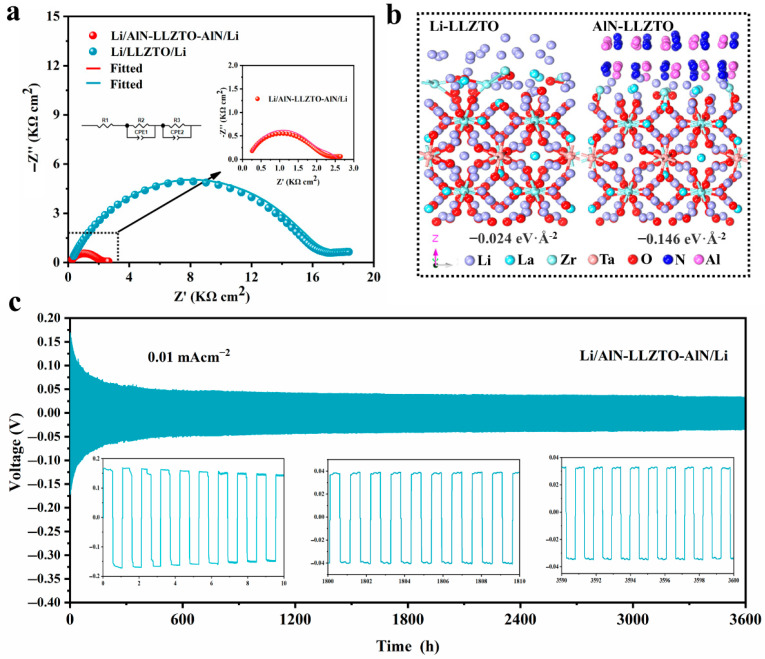
(**a**) Comparison of Nyquist plots of Li/AlN-LLZTO-AlN/Li and Li/LLZTO/Li at 30 °C; (**b**) DFT calculations of the interface formation energies for Li/LLZTO and AlN/LLZTO; (**c**) voltage profiles and details for the symmetric (Li/AlN-LLZTO-AlN/Li) cell for current densities of 0.01 mA cm^−2^ with 0.005 mAh cm^−2^.

To evaluate the effect of the AlN film on the contact at the interface and the cycle stability, both the symmetric Li/AlN-LLZTO-AlN/Li cells and Li/LLZTO/Li cells were assembled using the same method. As shown in Figure 4a, the Nyquist plots for the symmetric cell of non-modified LLZTO and AlN-LLZTO exhibit one semicircle. This semicircle is assigned to the interface resistance. Considering the symmetry of the Li/LLZTO interfaces, the interface resistance of a single Li/LLZTO interface is 7.89 kΩ cm^2^, while the interface resistance of LLZTO coated with AlN is reduced to 0.89 kΩ cm^2^. To better understand this effect, the interaction of Li/LLZTO, Li/AlN, and AlN/LLZTO was studied using density functional theory (DFT) calculations. As shown in Figure 4b and Figure S6, the interface formation energy of Li/LLZTO, AlN/LLZTO and Li/AlN are −0.024, −0.146, and −2.333 eV·Å^−2^, respectively, implying the intrinsic capabilities of AlN to wet LLZTO. Additionally, AlN has the intrinsic ability to wet Li, and the mutually wettable interface facilitates the diffusion of ions. The AlN film can ensure tight contact of the Li/LLZTO interface and promote Li-ion transport. Furthermore, the critical current density (CCD) is an important indicator to determine whether the electrolyte electrode system can work stably at high current-densities. In the system with a Li electrode and the garnet solid electrolyte, the CCD reflects the barrier between the solid electrolyte and Li dendrites at high current densities [61]. The Li/AlN-LLZTO-AlN/Li cells were measured at 30 °C, with a current density increase of 0.0625 mA cm^−2^ every 0.5 h from 0.12 mA cm^−2^ to 1.12 mA cm^−2^ for constant-current cycling. The solid line (red) in S7 indicates a sharp voltage fluctuation, around 0.4 mA cm^−2^, from which it can be identified as a CCD of 0.4 mA cm^−2^. However, the Li/LLZTO/Li cell was short-circuited at the beginning. The cycle performance of the symmetric Li-electrode cells was measured using galvanostatic charging and discharging (GCD) tests with a constant current at 30 °C. S8 shows that the Li/LLZTO/Li cell cannot cycle stably, and it short-circuits quickly due to the poor solid–solid contact interface. On the other hand, the Li/AlN-LLZTO-AlN/Li symmetric cell, after the introduction of AlN film, can cycle stably for more than 3600 h at 0.01 mA cm^−2^ (Figure 4c). This indicates that the introduction of the AlN film can improve the Li-electrode/electrolyte interface contact and facilitate the Li-ion transfer between the interfaces.

**Figure 5:**
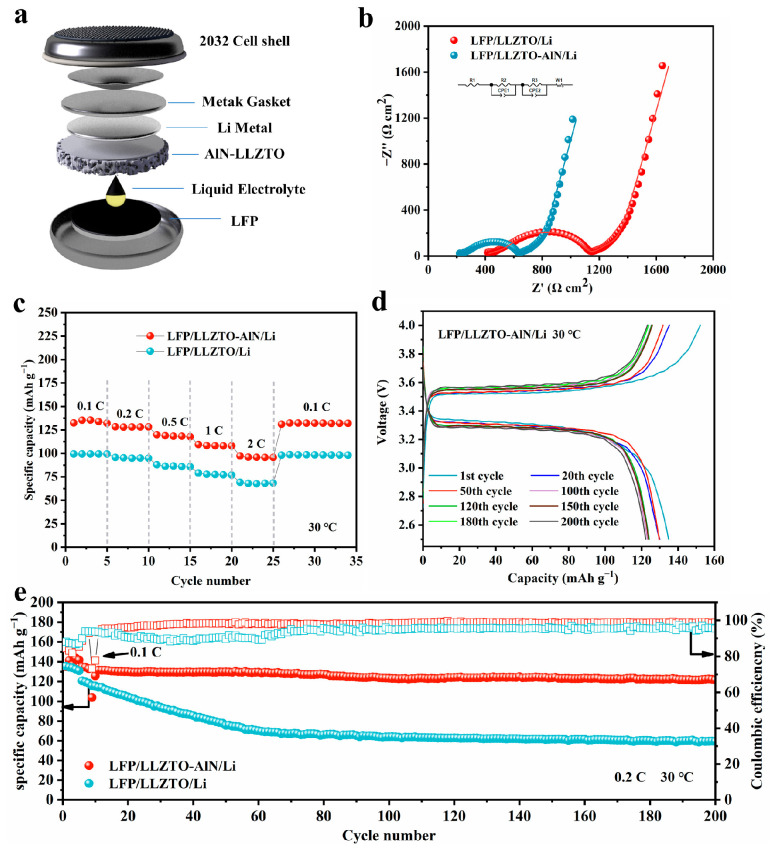
(**a**) Schematic configuration of the Li/AlN-LLZTO/LFP cell; (**b**) comparison of the EIS profiles of the cells using LLZTO with and without AlN modification; (**c**) rate performance of the Li/AlN-LLZTO/LFP cell; (**d**) the charge- and discharge-platform information for different cycles at 0.2 C; (**e**) long-term electrochemical performance of the Li/LLZTO/LFP, and the Li/AlN-LLZTO/LFP cell at 0.2 C.

To confirm the availability of AlN-modified LLZTO in a lithium-ion battery, both Li/AlN- LLZTO/LFP and Li/LLZTO/LFP hybrid solid-state full cells were assembled and tested. Figure 5a shows a schematic diagram of the structure of the hybrid solid-state full cell. A tiny amount of the liquid electrolyte was introduced between the LiFePO_4_ cathode and LLZTO electrolyte to wet the two-phase interface. Details of the preparation and assembly of the complete cell are provided in the Experimental section. The Nyquist plots of the hybrid solid-state Li/AlN-LLZTO/LFP and Li/LLZTO/LFP full cell were recorded. As shown in Figure 5b, each plot has a suppressed semi-circle in the high and medium frequency and a line in the low frequency. The turning points of the Li/LLZTO/LFP appear at ~1.47 Hz, where the values of the Z’-axis were evaluated with respect to the specific resistance of the total area (1010 Ω cm^2^). The specific resistance of the total area dropped to 640 Ω cm^2^ after the AlN film was introduced. Figure 5c shows the rate performance of the Li/AlN-LLZTO/LFP and Li/LLZTO/LFP cell. The discharge capacities of Li/AlN-LLZTO/LFP are 132.4, 128.4, 119.9, 109.4 and 97.2 mA h g^−1^ at 0.1, 0.2, 0.5, 1 C and 2 C, respectively. The rate performance of Li/AlN-LLZTO/LFP is significantly better than that of Li/LLZTO/LFP. After high-rate cycling, the Li/AlN-LLZTO/LFP recovers a discharge capacity of 131.1 mAh g^−1^ at 0.1 C. Figure 5d and Figure S9 show that the Li/AlN-LLZTO/LFP cell has the same charging and discharging platform as the typical LFP batteries that use a liquid electrolyte. Figure 5e (refer to S10 for details) shows the long-term cycling stability of the Li/AlN-LLZTO/LFP and Li/LLZTO/LFP cell at 0.2 C. As shown in Figure 5e, the Li/LLZTO/LFP cell has an initial charge and discharge capacity of 134.1 and 120.7 mAh g^−^^1^ with 90.0% coulomb efficiency, which is due to the poor interface contact between Li and the electrolyte. The cell-capacity decay-rate is fast during the cycle, and the capacity retention rate after 200 turns is only 49.3%. The cell, which is equipped with an AlN film, has an initial charge/discharge capacity of 144.7 and 134.5 mAh g^−1^, with a Coulombic efficiency of 92.9%. The Coulombic efficiency was ~99% during the cycling, and the discharge specific capacity of the cell still had 122.6 mAh g^−1^ after 200 cycles, with a capacity retention rate of 93.1%. It can be seen that the cell with the AlN-modified layer has both a better rate performance and cycle performance than the cell without interface modification. 

**Figure 6:**
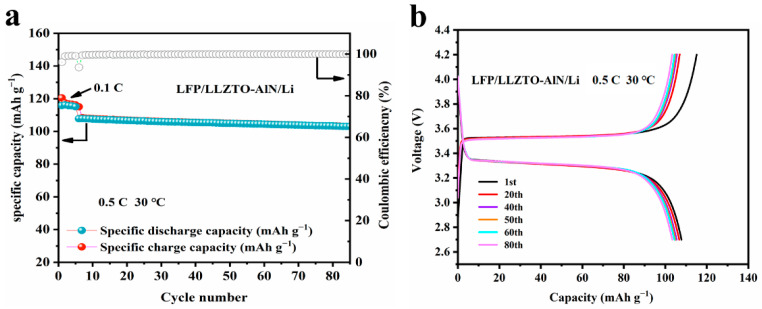
(**a**) Cycle performance of the Li/AlN-LLZTO/LFP cell at 0.5 C current density; (**b**) charge/discharge plateau of the Li/AlN-LLZTO/LFP at 0.5 C.

The Li/AlN-LLZTO/LFP-containing AlN modified layer was further tested for cycling stability at high current-densities (at 0.5 C). As shown in Figure 6a, a current density of 0.1 C was used for initial activation during the initial 5 cycles, and it can be seen that the capacity of the battery is lower at high current-densities. The initial charge and discharge capacities of the Li/LLZTO/LFP battery at 0.5 C were 116.2 mAh g^−1^ and 115.1 mAh g^−1^, respectively, and the coulombic efficiency during cycling was close to 100%. The specific discharge capacity of the battery was still 103.4 mAh g^−1^ after 80 cycles, with a capacity retention rate of 89.8%. Figure 6b shows the charging and discharging plateaus of the battery for different numbers of cycles. The results indicate that the introduction of the AlN layer enables the construction of a stable Li-electrode/electrolyte interface, which facilitates the transport of Li-ions between Li electrode and electrolyte and improves the performance of the full cells.

## 4. Conclusions

In this study, we synthesized cubic-phase LLZTO using a secondary sintering method and proposed an effective method to improve the wettability for the solid electrolyte of LLZTO on lithium, using an AlN interlayer. Theoretical calculations show that AlN has a high affinity for Li, while AlN is also wettable for LLZTO. The mutually wettable interface facilitates the diffusion of ions. The AlN film can ensure tight contact of the Li-metal/LLZTO interface and promote Li-ion transport. The assembled symmetric Li cells can cycle stably for 3600 h. The hybrid solid-state battery with the modified layer, which was assembled using LiFePO_4_ (LFP), still had a capacity of 120 mAh g^−1^ after 200 cycles and a capacity retention rate of 98%. This suggests a satisfactory capacity and excellent cycle stability. This also shows that the introduction of an AlN interlayer is a very effective method to produce a stable Li-electrode/solid electrolyte interface and to improve the cycling stability of the battery.

## Data Availability

Data presented in this article are available on request from the corresponding author.

## Supplementary Materials

The following supporting information can be downloaded at: https://www.mdpi.com/article/10.3390/nano12122023/s1, Figure S1: The X-ray characteristics of AlN as a commercial reactant. Figure S2: Cross-sectional SEM image of the LLZTO pellet. Figure S3: The Nyquist curves of LLZTO at 25 °C. Figure S4: EIS spectra of LLZTO pellets with Ag as blocking electrodes at different temperatures in the range 25~60 °C. The insets show the equivalent circuit for thus obtained EIS results. Figure S5: DFT calculations of interfacial formation energies of Li/LLZTO Li/AlN and AlN/LLZTO. Figure S6: (a) Critical current density of Li/ LLZTO/Li (b) Critical current density of Li/AlN-LLZTO/Li. Figure S7: Voltage profiles and details for the Li/LLZTO/Li symmetric cell at current densities of 0.01 mA cm^−2^ with 0.005 mAh cm^−2^. Figure S8: The charge and discharge platform information under different cycles at 0.2 C. Figure S9: (a) The long-term electrochemical performance of the Li/LLZTO/LFP cell under 0.2 C; (b) The long-term electrochemical performance of the Li/AlN-LLZTO/LFP cell under 0.2 C. Table S1: The conductivity data and resistances of LLZTO pellets with Ag as blocking electrodes at different temperatures in the range 25~60 °C. Table S2: Nyquist plots fitted data for lithium symmetric cells. Table S3: Nyquist plots fitted data for hybrid solid-state full cells.
