# Supplementary material for: Improvement of the Interface between the Lithium Anode and a Garnet-Type Solid Electrolyte of Lithium Batteries Using an Aluminum-Nitride Layer
Source: Nanomaterials (Basel). 2022 Jun 12;12(12):2023. doi: 10.3390/nano12122023 (PMC9227169; doi:10.3390/nano12122023)
Supplement: Supplementary file 1 [file nanomaterials-12-02023-s001.zip › nanomaterials-1737180-supplementary.pdf]

Improvement of the Interface between the Lithium Anode and a Garnet-Type Solid Electrolyte of Lithium Batteries Using an Aluminum-Nitride Layer

Wen Jiang [1], Lingling Dong [1], Shuanghui Liu [1], Bing Ai [1], Shuangshuang Zhao [2], Weimin Zhang [1], Kefeng Pan [1,*] and Lipeng Zhang [2,*]

1 School of Chemistry and Chemical Engineering, Shandong University of Technology, Zibo 255049, China; wengejang@126.com (W.J.); donglingling202107@163.com (L.D.); shuanghuiliu2020@163.com (S.L.); aibing@sdut.edu.cn (B.A.); wmzhang@sdut.edu.cn (W.Z.)
2 School of Materials and New Energy, South China Normal University, Shanwei 516600, China; 20219207@m.scnu.edu.cn
* Correspondence: xiaopandy@126.com (K.P.); zhanglipeng@sdut.edu.cn (L.Z.)

S1 The X-ray characteristics of AlN as a commercial reactant

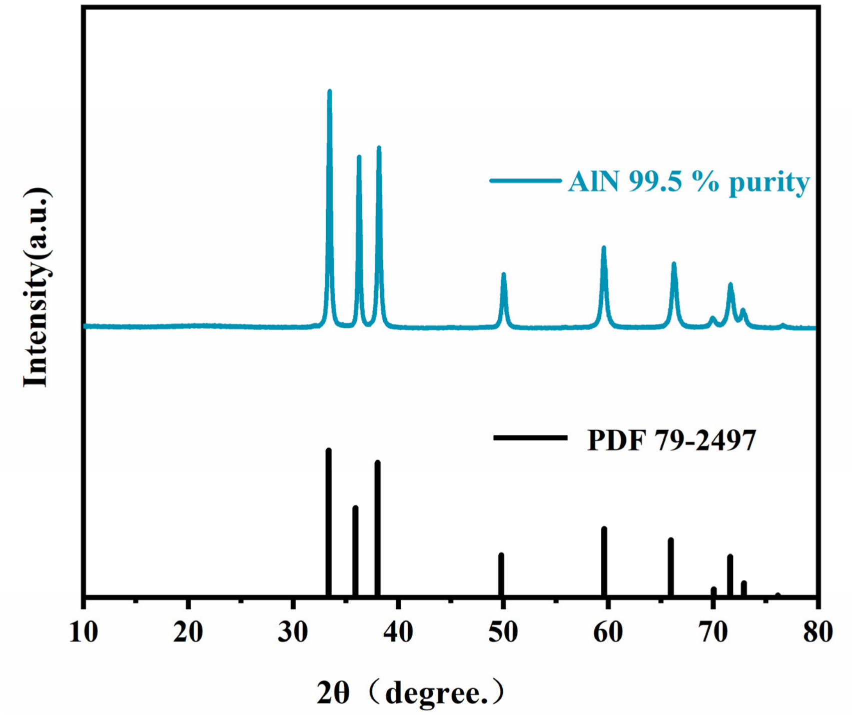

Figure S1. The X-ray characteristics of AlN as a commercial reactant

S2 DFT Method

The interface models of Li (0 0 1)/LLZTO (0 0 1), Li (0 0 1)/AlN (0 0 1) and AlN (0 0 1)/LLZTO (0 0 1) were constructed by Li (0 0 1) slab, AlN (0 0 1) and LLZTO(0 0 1).To solve the crystal lattice match problem, the lattice parameters of a and b of Li (0 0 1) 3×3 surface slab was set the same as that of AlN (0 0 1) 5×5 surface slab; Li (0 0 1) 3×3 surface slab and AlN (0 0 1) 5×5 surface slab are constructed to match the lattice parameter of LLZTO (0 0 1) 1×1 surface slab. The interface formation energies of Li (0 0 1)/LLZTO (0 0 1), Li (0 0 1)/AlN(001) and AlN (0 0 1)/LLZTO (0 0 1) were evaluated by energy difference between an

interface system and the bulk energy of the two materials that comprise it, i.e., $E_{IFE} = (E_{ab} – N_a*E_a – N_b*E_b)/2S$, where Eab denotes the total energy of the complete system containing the interface, and depends on how many formula units of materials a and b comprise the interface (N_a and N_b, respectively), E_a and E_b are the bulk energy per formula unit for materials a and b, respectively, and S refers to the interfacial area, 2 means two interfaces in the interface models.

S3 Cross -sectional SEM image of the LLZTO pellet

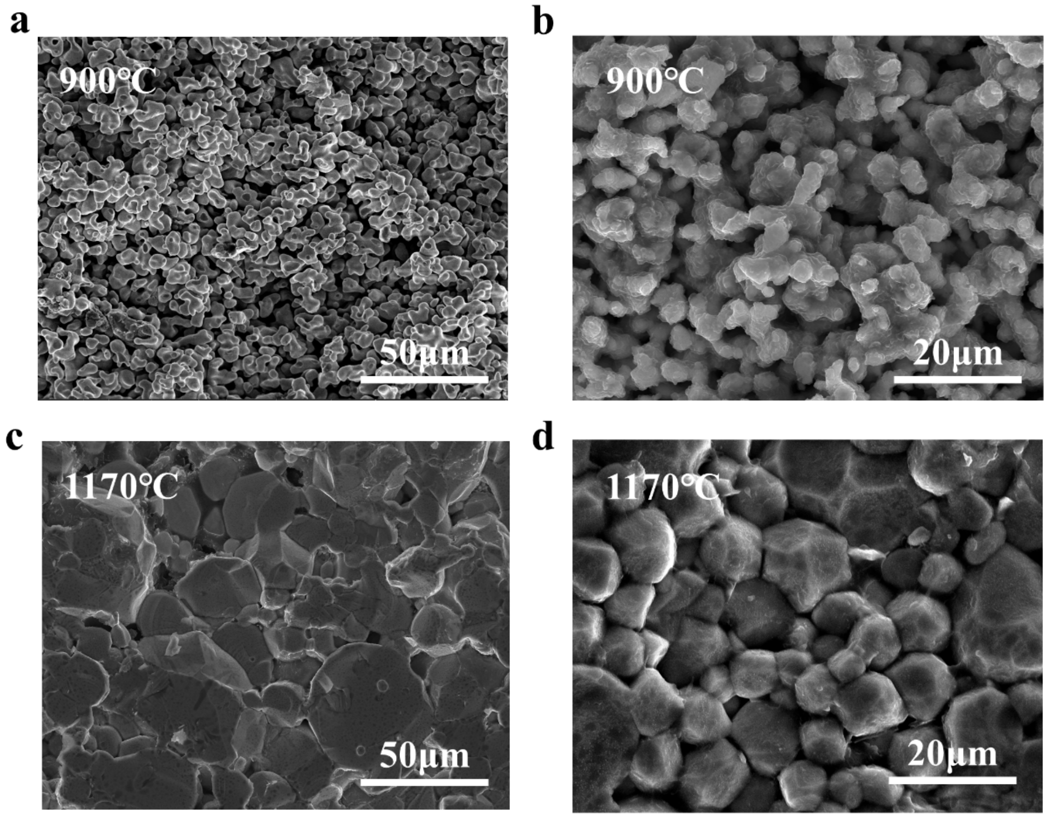

Figure S2. Cross -sectional SEM image of the LLZTO pellet (a) (b) 900 ℃; (c) (d) 1170 ℃.

S4 The Nyquist curves of LLZTO at 25 °C

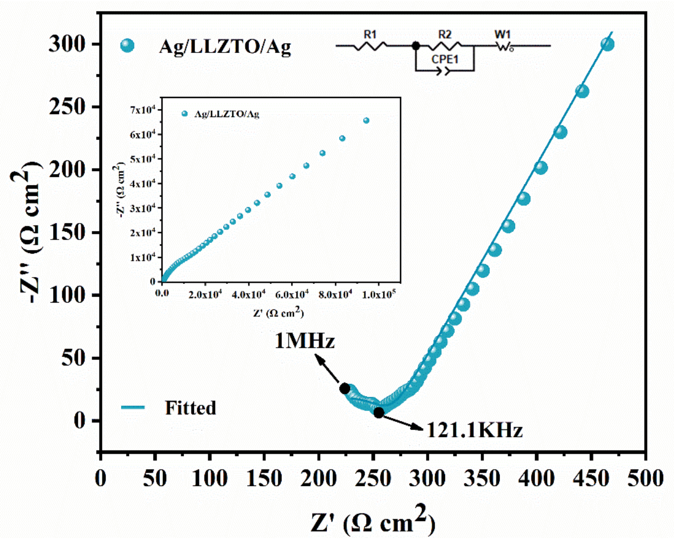

Figure S3. The Nyquist curves of LLZTO at 25 °C. The insets show the equivalent circuit for thus obtained EIS results.

NOTICE: the σ (ionic conductivity) of LLZTO was calculated by:

$$\sigma = \frac{L}{RS}$$

Where the σ is ionic conductivity (S cm^{-1}), L is thickness(cm), R is resistance(Ω), S is active area(cm^2). the thickness of all electrolytes is controlled at 0.8 mm, and the diameter of the lithium foils is 10 mm.

S5 Arrhenius equation

The activation energies (Ea) of LLZTO for conductivity can be calculated according to Arrhenius equation σ(T) = Aexp(−Ea/RT), where k is the Boltzmann's constant, T is the temperature (K) and A is a pre-exponential factor.

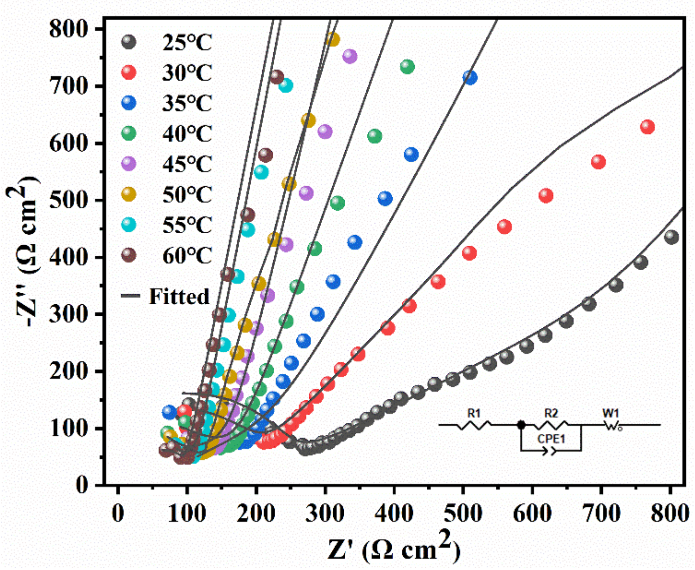

Figure S4. EIS spectra of LLZTO pellets with Ag as blocking electrodes at different temperatures in the range 25°C~60°C. The insets show the equivalent circuit for thus obtained EIS results.

Table S1. The conductivity data and resistances of LLZTO pellets with Ag as blocking electrodes at different temperatures in the range 25℃~60℃.

Temp. (℃)	R (Ω)	σ（x10^{-4} S·cm^{-1}）	1000·T^{-1} K^{-1}	LnσT (S·cm^{-1}·K)
25	264.8	2.70	3.35	−2.51
30	236.6	3.02	3.29	−2.38
35	222.7	3.21	3.24	−2.31
40	193.4	3.70	3.19	−2.15
45	139.3	5.13	3.14	−1.81
50	126.46	5.66	3.09	−1.69
55	108.56	6.59	3.04	−1.53
60	100.92	7.09	3.00	−1.44

S6 DFT calculations of interfacial formation energies of Li/LLZTO Li/AlN and AlN/LLZTO.

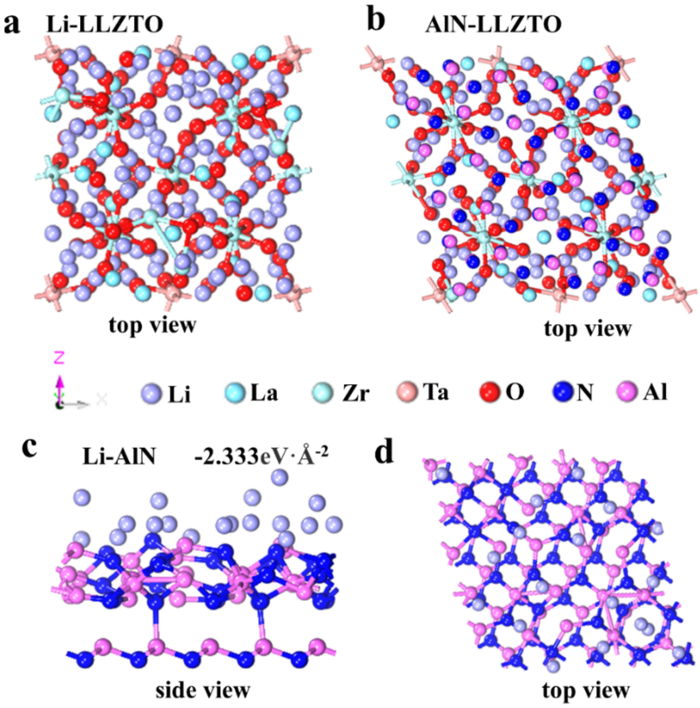

Figure S5. DFT calculations of interfacial formation energies of (a) Li/LLZTO, (b) AlN/LLZTO and (c) (d) Li/AlN.

Table S2. Nyquist plots fitted data for lithium symmetric cells

Cells	R_1 (Ω)	R_2 (Ω)	R_3 (Ω)
Li/LLZTO/Li	148.8	15003	3743
Li/AlN-LLZTO-AlN/Li	177.1	1608	824

NOTICE: R_1, R_2, and R_3 represent the bulk resistance of electrolyte, the interfacial resistance, and the charge transfer resistance, respectively.

S7 Critical current density (CCD) determination by galvanostatic cycling from 0.12 mA cm^{-2} to 1.12 mA cm^{-2} with increasing current at 0.0625 mA cm^{-2} intervals at 30°C.

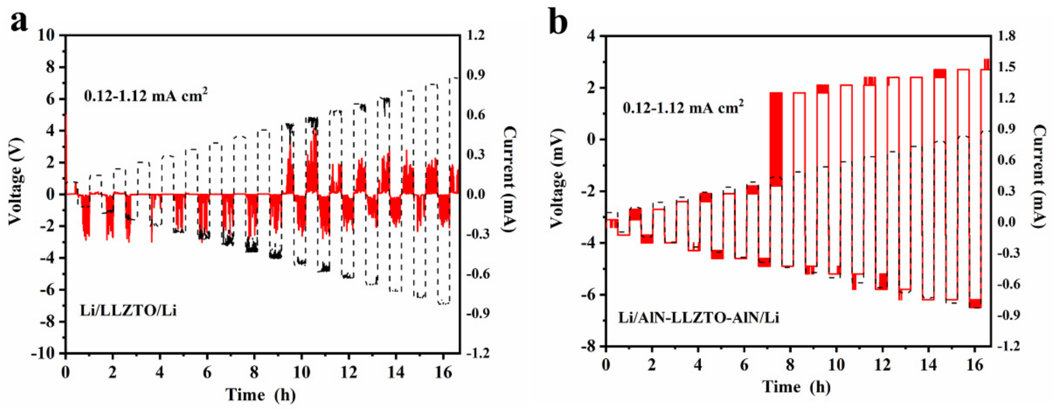

Figure S6. (a) Critical current density of Li/ LLZTO / Li (b) Critical current density of Li/AlN-LLZTO/ Li.

S8 Voltage profiles and details for the Li /LLZTO / Li symmetric cell at current densities of 0.01mA cm^{-2} with 0.005mAh cm^{-2}.

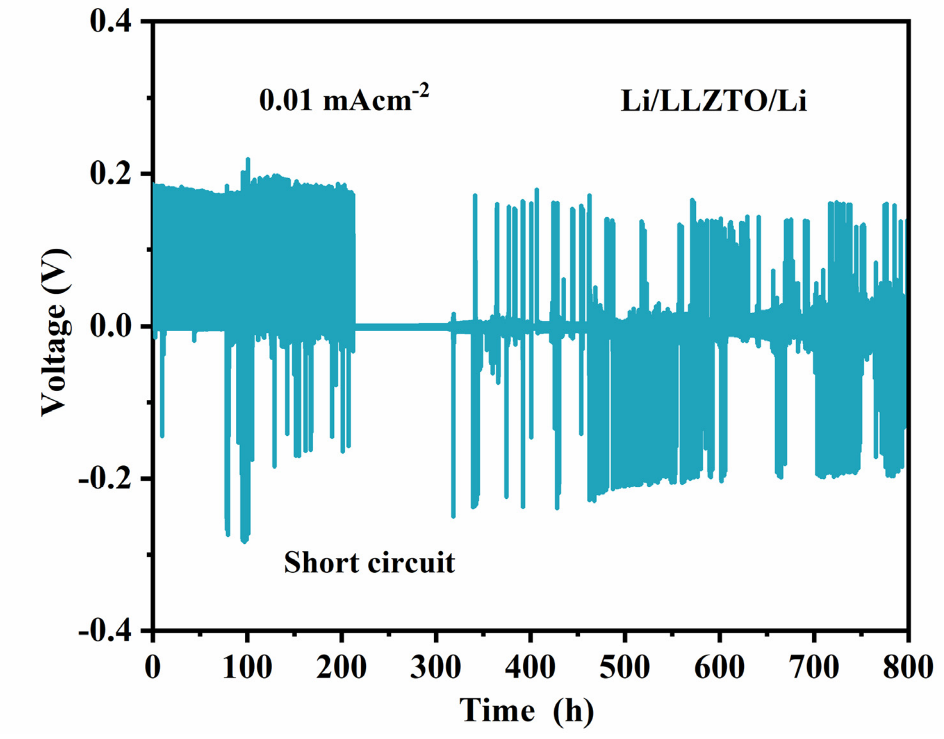

Figure S7. Voltage profiles and details for the Li /LLZTO / Li symmetric cell at current densities of 0.01mA cm^{-2} with 0.005mAh cm^{-2}.

S9 The charge and discharge platform information under different cycles at 0.2C.

Table S3. Nyquist plots fitted data for hybrid solid state full cells

Cells	R_1 (Ω)	R_2(Ω)	R_3 (Ω)
Li/LLZTO/LFP	376. 4	216.3	527. 5
Li/AlN-LLZTO/LFP	205. 2	109.2	305. 4

NOTICE: R_1, R_2 and R_3 represent the bulk resistance of electrolyte, the interfacial resistance, and the charge transfer resistance, respectively.

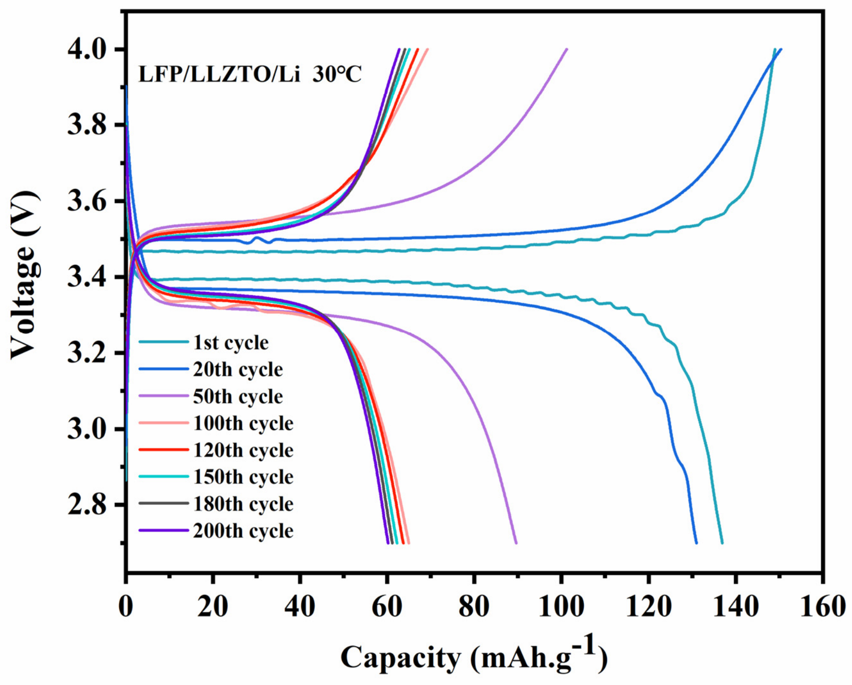

Figure S8. The charge and discharge platform information under different cycles at 0.2C.

S10 The long-term electrochemical performance of the Li /LLZTO /LFP and the Li /AlN-LLZTO /LFP cell under 0.2C.

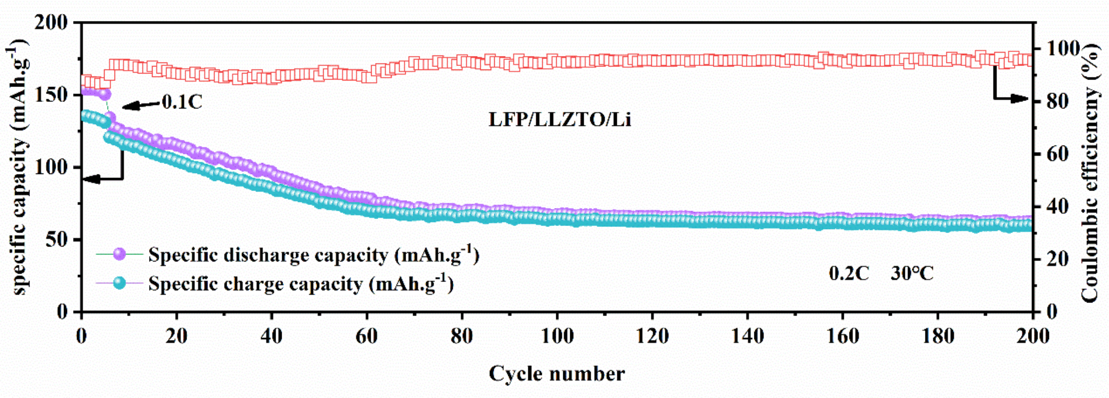

Fig S9. (a)The long-term electrochemical performance of the Li /LLZTO /LFP cell under 0.2C.

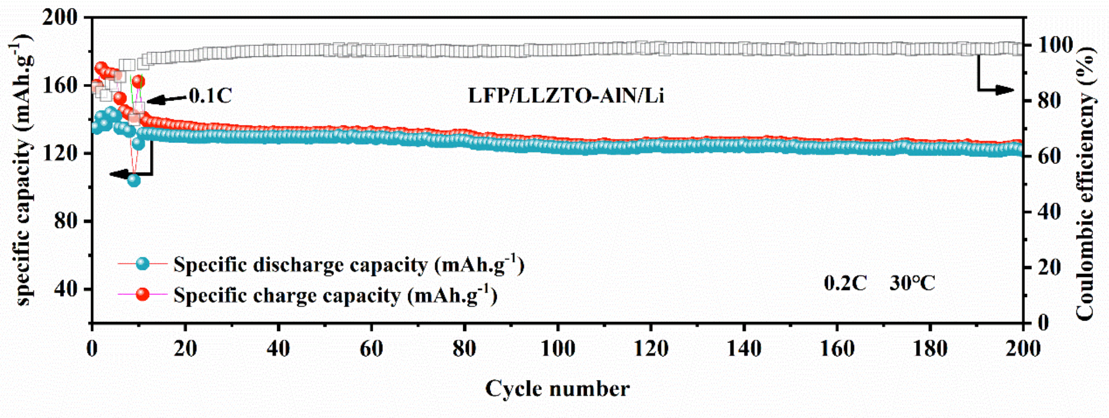

Figure S9. (b)The long-term electrochemical performance of the Li /AlN-LLZTO /LFP cell under 0.2C.
